# Exacerbation of Subthreshold PTSD Symptoms in a Great East Japan Earthquake Survivor in the Context of the COVID-19 Pandemic

**DOI:** 10.1155/2021/6699775

**Published:** 2021-02-10

**Authors:** Arinobu Hori, Toyoaki Sawano, Akihiko Ozaki, Masaharu Tsubokura

**Affiliations:** ^1^Department of Psychiatry, Hori Mental Clinic, Minamisoma, Fukushima, Japan; ^2^Department of Surgery, Jyoban Hospital of Tokiwa Foundation, Iwaki, Fukushima, Japan; ^3^Department of Radiation Health Management, Fukushima Medical University School of Medicine, Fukushima, Fukushima, Japan; ^4^Department of Breast Surgery, Jyoban Hospital of Tokiwa Foundation, Iwaki, Fukushima, Japan

## Abstract

**Background:**

In 2011, the people of Fukushima, Japan, experienced the Great East Japan Earthquake (GEJE), a complex disaster of earthquake, tsunami, and nuclear accident. Its residents are experiencing a second global disaster, a COVID-19 pandemic in 2020.

**Objective:**

In this article, we aimed at discussing the effects of subthreshold PTSD in a previous disaster on an exacerbation of PTSD symptoms in another disaster.

**Method:**

We present a case of subthreshold PTSD in the context of a nuclear accident and exacerbation of symptoms due to the COVID-19 pandemic.

**Results:**

Exacerbation of subthreshold PTSD symptoms was likely due to the reemergence of an urgent atmosphere similar to the previously experienced traumatic event.

**Conclusions:**

PTSD may occur not only in those who experience the actual life-threatening like ICU admission but in those who experience the atmospheric change of society. This case demonstrated the characteristics of subthreshold PTSD caused by two disasters that shared a similar sense of insecurity, the scale of impact on the society, invisibility of the threat, restricted movement, and authoritative conflicts. These commonalities led to a recurrence and exacerbation of initial symptoms. This finding should be shared with those involved in the care system for victims' mental health suffering from a large-scale disaster, and we need further research about the issue.

## 1. Introduction

Large-scale calamities, such as the nuclear disaster that followed the Great East Japan Earthquake (GEJE) and the coronavirus disease (COVID-19) pandemic, have an overwhelming impact on society at large and are accompanied by an individual's sense of insecurity owing to the invisibility of the threat, limitation of freedom and outdoor movement, and conflict between government authorities and experts.

Correspondingly, posttraumatic stress disorder (PTSD) was found to have increased among those who affected by the GEJE and was reported to be a serious health consequence for these survivors [1, 2]. Even at subthreshold levels, PTSD symptoms could lead to long-term declines in social functioning [3, 4]. Despite these adverse effects, subthreshold PTSD might go underdiagnosed and consequently undertreated even long after treatment for other noticeable psychiatric problems like mood disorders has begun, due to clinician's lack of consideration and underreporting of trauma by patients. This tendency may be exacerbated by the absence of core symptoms of PTSD, such as reexperiencing traumatic memories during nightmares [5]. Furthermore, cases of subthreshold PTSD are more likely to follow a difficult course and may be misinterpreted as the exact origin of the chronicity factor in the aggressive behaviors of patients [4].

Yet, the actual conditions of subthreshold PTSD [3, 4] are scarcely understood. Moreover, reports of having experienced two global disasters in a few years are rare. Thus, we discuss the case of a GEJE survivor, who was initially treated for a chronic depressive disorder but later developed severe insomnia associated with an intense fear triggered by the spread of COVID-19 in his hometown.

## 2. Case Presentation

The patient was divorced before the earthquake and lived alone. In his late 40s, he experienced the GEJE and subsequent nuclear power plant accident in 2011. Although evacuation orders were issued in neighboring districts during the nuclear disaster, the patient remained in his residence area and continued to go to work. He reported that he had considered moving out but chose to stay due to his strong emotional attachment to the area. As people evacuated and the town's population rapidly dwindled, the patient reported becoming aware of overwhelming feelings of loneliness and anxiety. He admitted later that a traumatic memory for him was the days he spent in fear of radiation exposure after the nuclear accident. About a month after the GEJE, he took a job in another area at the direction of his company, where he sometimes felt that people around him were avoiding him. He felt very hurt when this happened. At his company's direction, he returned to his hometown in a month.

Two years after the GEJE, the patient reported experiencing headaches and insomnia but did not seek help. Three years later, in 2016, he visited our mental health clinic with symptoms of anxiety, depressed mood, insomnia, and decreased motivation. We confirmed that he was in his hometown when the earthquake and the nuclear accident happened, but he did not experience the tsunami up close nor get involved in searching for the victims' bodies. Furthermore, he never expressed his fears about the disaster at this time, rather his frustration that the company did not fully recognize and reward his contributions to the company during the disaster. He reported that his most significant stressor was his relationship with his boss. The patient was diagnosed with major depressive disorder [5] and was started on a medication regime that primarily consisted of serotonin selective reuptake inhibitors. We missed his symptoms of PTSD.

The treatment was only partially effective since symptoms were exacerbated by stress. The patient sometimes aggressively criticized his company, gradually took more time off from work, and eventually left the company. Our efforts to increase his awareness about self-care were unsuccessful. He mostly focused on the company's problems and seldom spoke about his memory of the disaster during medical interviews.

In April 2020, when cases of COVID-19 emerged in the patient's neighborhood, he reported that he felt scared because daily calls for restraint and television updates on COVID-19 triggered memories of the GEJE. He further reported that he had been experiencing severe insomnia for more than a month, and we had started him on antipsychotic medication (olanzapine 5 mg), with partial effect. People in his hometown usually lacked knowledge about mental illnesses such as PTSD and considered mental illness as shame and avoided confronting it. Therefore, when confronted about his avoidance of discussing the GEJE during his treatment, he finally reported feeling ashamed to talk about his fears. After talking about his traumatic memories concerning GEJE and the nuclear accident, his symptoms were partially relieved, and he continued his outpatient treatment.

At this occasion, our explanation convinced him, when he was not aware of the trauma's effects during the disaster. He was satisfied that we finally recognized his suffering and labor during the disaster. It has not prevailed that the town's atmosphere in marginal situations such as the nuclear accident or COVID-19 pandemic can cause PTSD. When we discussed with him the idea of making an academic case report on it, he gave his positive consent.

## 3. Discussion

Based on reported symptoms, it can be surmised that the patient experienced the GEJE as traumatic. However, he did not realize that his mental problems caused his nonfunctional state. He thought it was due to physical problems such as headaches and insomnia or anger at the company for not recognizing his contribution to the disaster. Then, psychiatric treatment started just in 2016, five years after the GEJE. When psychiatric treatment began, we soon noticed the presence of depression. However, we missed PTSD symptoms because he had not experienced severe conditions of the disaster expressed in visuals and narratives, which would lead to typical PTSD, and his complaints centered on dissatisfaction with his company. Lack of social support and a dependent personality that made him attached to the stricken area soon after the disaster were considered factors contributing to his symptoms' chronicity.

We finally recognized the existence of PTSD four more years later, in 2020, when the threat of the COVID-19 pandemic became apparent. On this occasion, a detailed interview revealed that the patient had avoidance of trauma-related stimuli after the disaster, negative alterations in cognitions and mood, and trauma-related arousal and reactivity. Furthermore, based on the following considerations, his aggressive behavior towards his company could be interpreted as a symptom of subthreshold PTSD [6]. However, the patient did not report any intrusive symptoms typical of PTSD, even in this situation. So, how should we think about the lack of intrusive symptoms? The patient experienced a major earthquake and nuclear accident in 2011, but the experience did not appear visually traumatic. He did not get caught in the tsunami that followed the earthquake either. However, traumatic memories are not only limited to visual images nor event narratives. For example, a bodily sensation of shaking at high altitudes could be the core element of the traumatic experience, as described in a case associated with the GEJE [7]. Similarly, the traumatic memory in this case was associated with the town's urgent atmosphere after the nuclear accident.

Despite a lack of corroborating evidence and expert opinion, popular media frequently reported adverse effects of radiation exposure due to the nuclear accident that triggered anxiety within the general population. The rural community where the current patient lived is generally characterized by very close-knit and interdependent relationships that incur mutual support and intrusive monitoring. Thus, those who disrupt the harmony of the community are faced with heavy criticism and blame. This kind of persecutory anxiety was prevalent in the affected area's urgent atmosphere after the nuclear accident and likely underlined our patient's traumatic fears.

Both radiation exposure from nuclear accidents and viral infections are invisible and pose a great threat to our health and life. The urgent atmosphere of the town, resulting from the COVID-19 outbreak was similar to that of the nuclear accident and acted as a trigger for the patient's traumatic reactions, namely, insomnia and impatience [5]. It is rare for a patient to experience a global disaster twice, and in this unique circumstance of the pandemic, subthreshold PTSD of the former nuclear accident has become evident.

Based on this case study, we propose the following recommendation of assessment and supporting strategy in the affected population in a large-scale disaster like GEJE or COVID-19 pandemic.

1. The involved professionals should be aware of the possibility that PTSD may occur not only in those who experience the actual life-threatening like ICU admission but in those who experience the atmospheric change of society during a disaster
2. Stakeholders should be aware that recognizing the horrors of a large-scale disaster can have a calming effect on patients' emotions; at the same time, lack of recognition can be highly frustrating for patients. On the contrary, we should also inform the community about the great psychological damage that an atmosphere of harsh condemnation of certain people could cause during a disaster
3. In Fukushima Prefecture, a remote support system has been established to care for the mental health of people who experienced the 2011 earthquake and nuclear accident [8]. The above two empirical findings should be shared with those involved in postdisaster systems like Fukushima's one, and we need further research about the issue

## Data Availability

No data were used to support this study.
